# Supplementary material for: Nano-Needle Boron-Doped Diamond Film with High Electrochemical Performance of Detecting Lead Ions
Source: Materials (Basel). 2023 Oct 31;16(21):6986. doi: 10.3390/ma16216986 (PMC10648918; doi:10.3390/ma16216986)
Supplement: Supplementary file 1 [file materials-16-06986-s001.zip › materials-2667416-supplementary.pdf]

Supplementary Materials

Article

Nano-Needle Boron-Doped Diamond Film with High Electrochemical Performance of Detecting Lead Ions

Xiaoxi Yuan [1,2], Mingchao Yang [3], Xu Wang [1], Yongfu Zhu [1,*] and Feng Yang [2,*]

[1] Key Laboratory of Automobile Materials, Ministry of Education, School of Materials Science and Engineering, Jilin University, Changchun 130022, China; xxyuan@jlenu.edu.cn (X.Y.)
[2] Institute for Interdisciplinary Quantum Information Technology, Jilin Engineering Normal University, Changchun 130052, China
[3] Department of Physics, Hebei Normal University of Science and Technology, Qinhuangdao 066000, China; yangmc17@mails.jlu.edu.cn
* Correspondence: yfzhu@jlu.edu.cn (Y.Z.); yangfeng@jlenu.edu.cn (F.Y.)

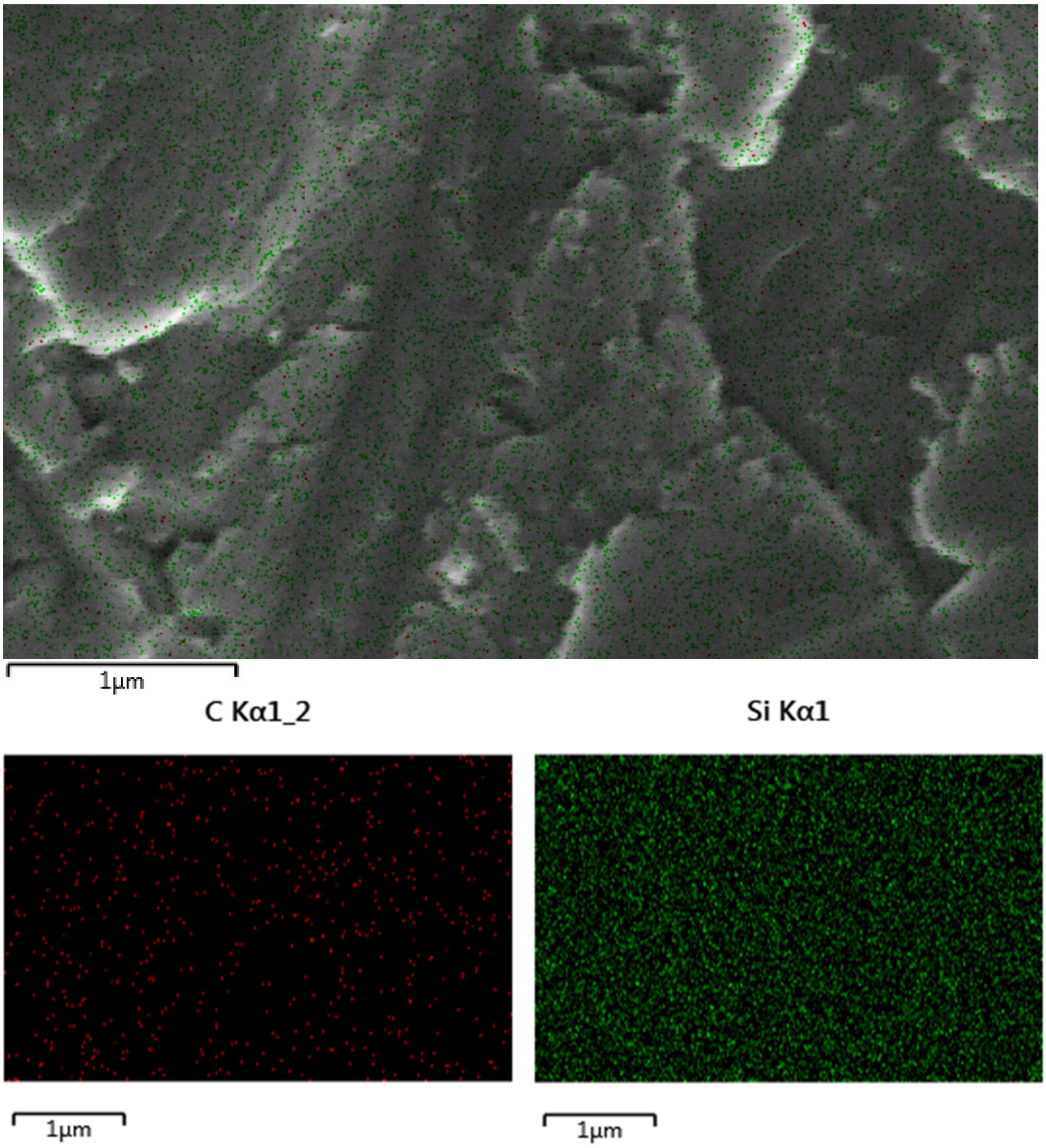

Figure S1. SEM and EDS images of the seeded Si substrates.

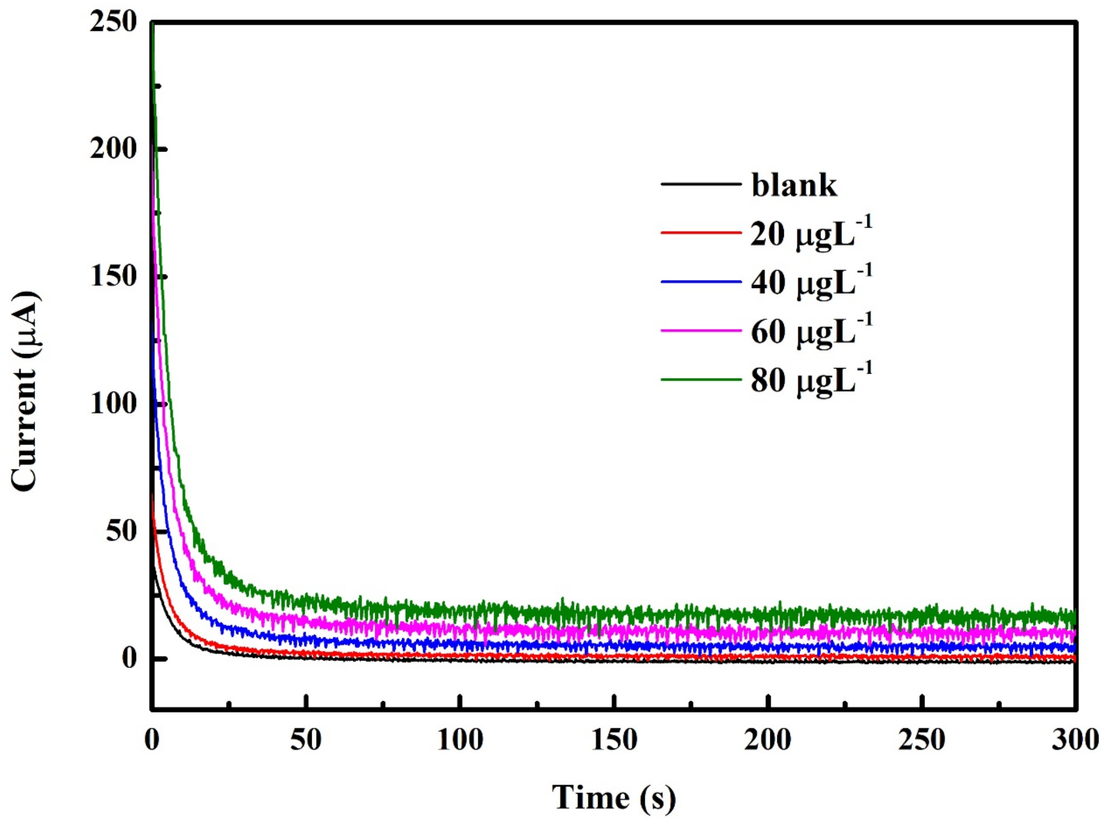

Figure S2. Chronoamperometry i-t curve at Pb^{2+} concentrations between 0 and 80 μgL^{-1}.

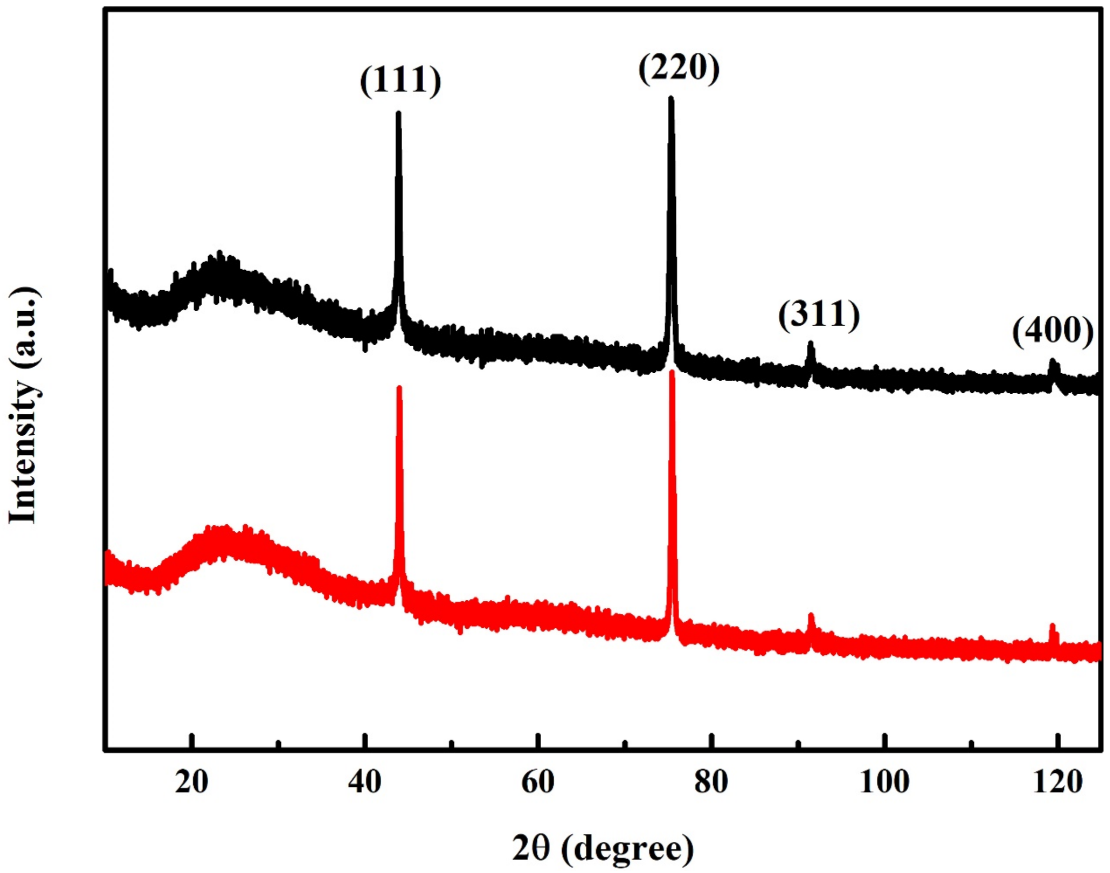

Figure S3. XRD of NNBDD before (black line) and after (red line) electrochemical sensing tests.
